# Supplementary material for: Comparative Genomics and Transcriptomics Analyses Reveal a Unique Environmental Adaptability of Vibrio fujianensis
Source: Microorganisms. 2020 Apr 13;8(4):555. doi: 10.3390/microorganisms8040555 (PMC7232310; doi:10.3390/microorganisms8040555)
Supplement: Supplementary file 1 [file microorganisms-08-00555-s001.pdf]

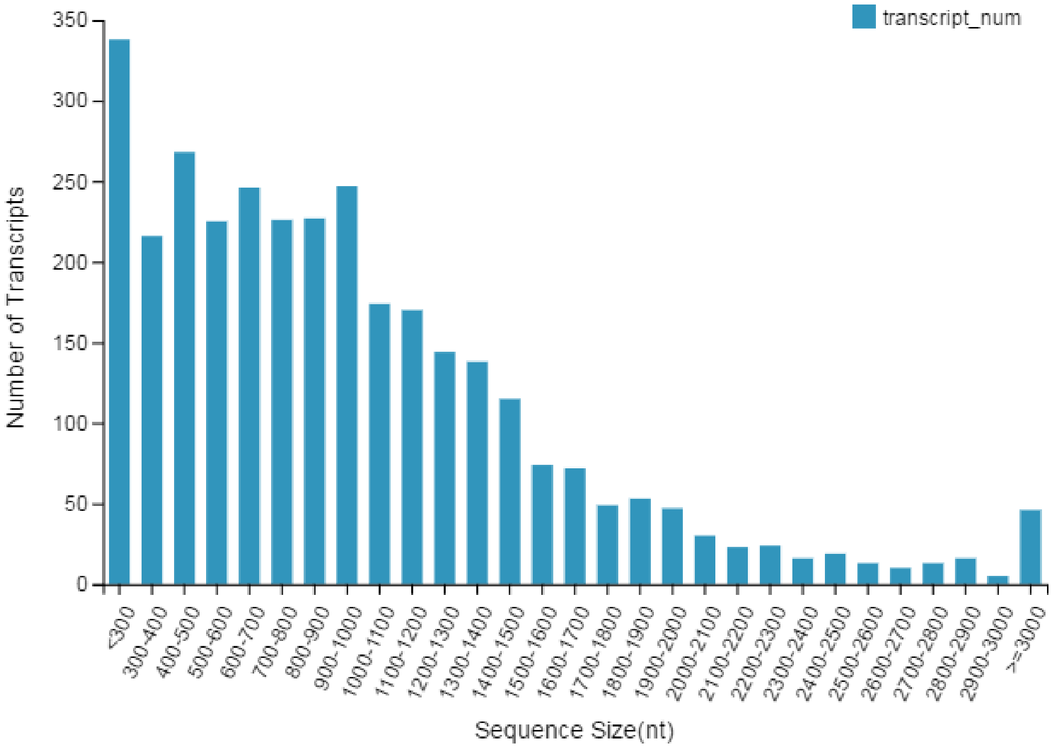

Figure S1. Sequence length distribution of *V. fujianensis* transcripts analyzed in this study.

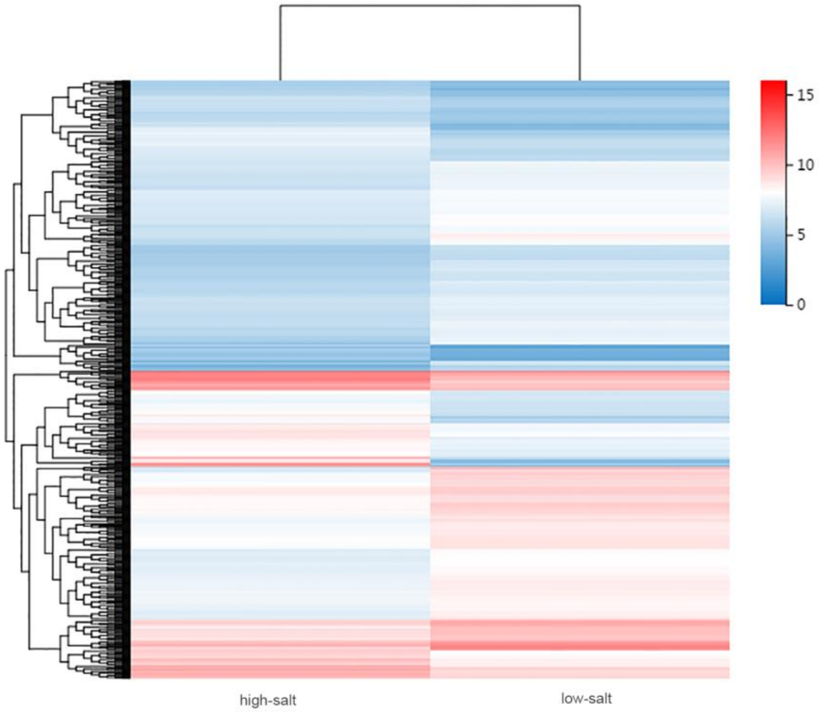

Figure S2. Differentially expressed genes (DEGs) cluster analysis of gene expression patterns between the high-salt and low-salt condition. The red color indicates high relative expression, and the blue color indicates low relative expression.

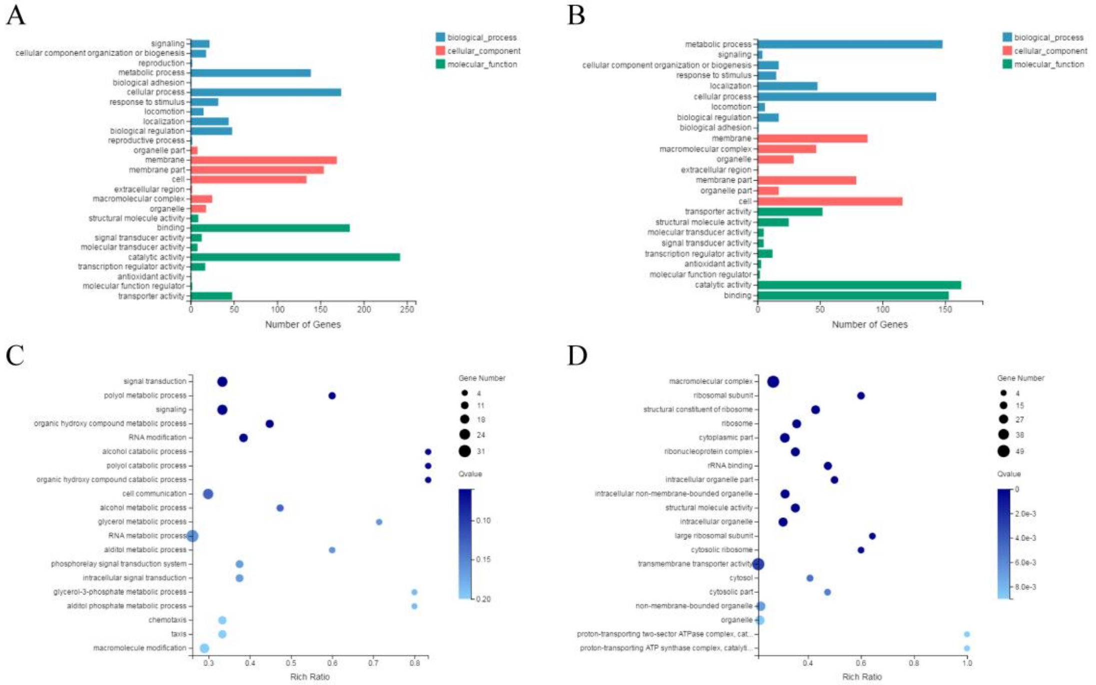

Figure S3. (**A**) Functional classification of gene ontology (GO) annotations of the up-regulated differentially expressed genes (DEGs). (**B**) Functional classification of GO annotations of the down-regulated DEGs. (**C**) Bubble charts of GO enrichment analysis of the up-regulated DEGs. (**D**) Bubble charts of GO enrichment analysis of the down-regulated DEGs. The x-axis corresponds to fold enrichment values while the y-axis indicates the top 20 GO enriched terms. Size of the bubble indicates the number of significant genes in the given enriched term.

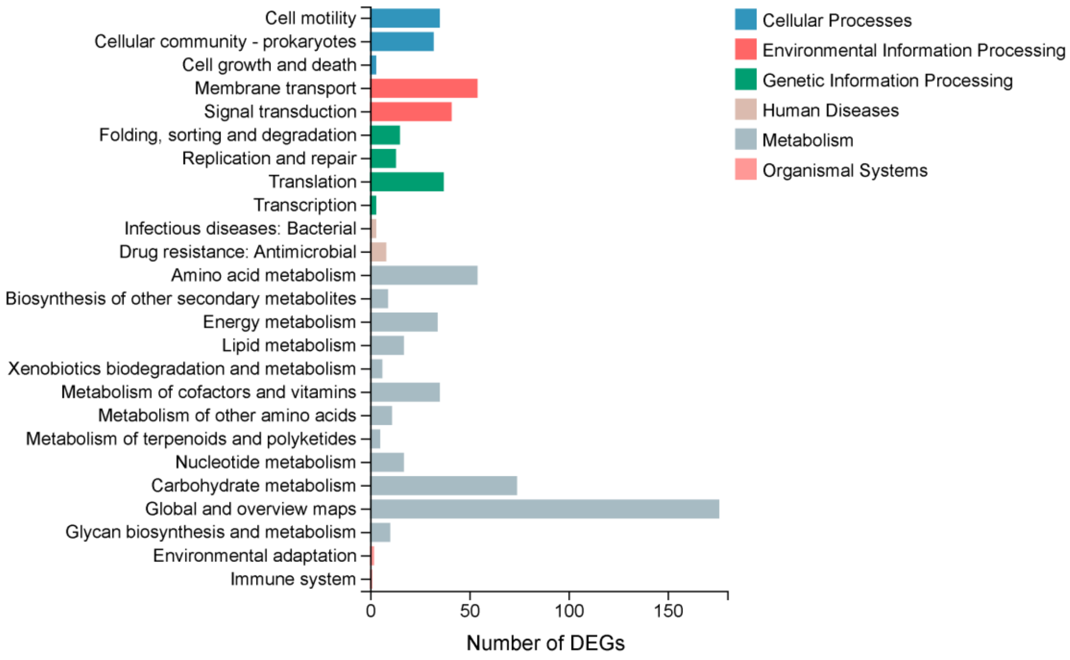

Figure S4. Functional classification of KEGG pathway of differentially expressed genes (DEGs). The y-axis indicates the name of the KEGG metabolic pathways. The x-axis indicates the number of the total DEGs annotated between high-salt and low-salt condition.

Table S1. Overview of 16S rRNA gene sequences of *Vibrio* species analyzed in this study.

No.	Species	Strain	Isolation Region	Isolation Source	Isolation Year	16S rRNA Accession
1	*Vibrio aerogenes*	ATCC 700797^{T}	China，Taiwan	Seagrass bed sediment	1993	AF124055
2	*Vibrio aestivus*	CECT 7558^{T}	Spain	Coastal seawater	2008	HE613734
3	*Vibrio aestuarianus*	ATCC 35048^{T}	USA	Oyster	-	X74689
4	*Vibrio agarivorans*	CECT 5085^{T}	Spain	Seawater	1996	AJ310647
5	*Vibrio albensis*	ATCC 14547	Elbe River	Fish	-	EF032499
6	*Vibrio alfacsensis*	CAIM 1831^{T}	Spain	Gill	2009	JF316656
7	*Vibrio alginolyticus*	ATCC 17749^{T}	Japan	Metamorphic horse mackerel	-	X56576
8	*Vibrio algivorus*	SA2^{T}	Japan	Turbo cornutus	2013	LC060680
9	*Vibrio anguillarum*	ATCC 19264^{T}	Norge	Sick squid	1956	X16895
10	*Vibrio antiquarius*	BV25Ex	USA	Oyster	2007	AF319769
11	*Vibrio aphrogenes*	CA-1004^{T}	Japan	Seawater	1994	KX713151
12	*Vibrio areninigrae*	KCTC 22122^{T}	South Korea	Soesaggak Beach, black sand	-	EU143360
13	*Vibrio artabrorum*	LMG 23865^{T}	Spain	Ruditapes philippinarum	2005	EF599164
14	*Vibrio astriarenae*	JCM 19233^{T}	Japan	Coral reef seawater	-	KP342514
15	*Vibrio atlanticus*	CECT 7223^{T}	Spain	Ruditapes philippinarum	2005	EF599163
16	*Vibrio atypicus*	HHS02^{T}	China	Penaeus chinensis	2007	FJ009624
17	*Vibrio azureus*	NBRC 104587^{T}	Japan	Seawater	2005	AB428897
18	*Vibrio barjaei*	3062^{T}	Spain	Ruditapes decussatus broodstock	2011	LN867554
19	*Vibrio bivalvicida*	605^{T}	Spain	Venerupis decussata	2012	HF568951
20	*Vibrio brasiliensis*	LMG 20546^{T}	Brazil	Scallop hatchery	1999	AJ316172
21	*Vibrio breoganii*	CECT 7222^{T}	Spain	Ruditapes decussatus	2004/2005	EF599161
22	*Vibrio campbellii*	ATCC 25920^{T}	USA	Seawater	1971	X56575
23	*Vibrio caribbeanicus*	DSM 23640^{T}	Netherlands	Scleritoderma cyanea (marine sponge)	2005	GU223601

24	*Vibrio casei*	WS 4539^{T}	Germany	Surface of French smear ripened cheese	2010	FJ968722
25	*Vibrio celticus*	LMG 23850^{T}	Spain	Ruditapes decussatus	2004	EF599162
26	*Vibrio chagasii*	LMG 21353^{T}	Norge	Gut of fish	1997	AJ316199
27	*Vibrio cholerae*	CECT 514^{T}	Asia	Cholera patient	-	X76337
28	*Vibrio cidicii*	2756-81^{T}	-	River water	1981	KJ807108
29	*Vibrio cincinnatiensis*	NCTC 12012^{T}	USA	Patient	-	X74698
30	*Vibrio comitans*	GHG2-1^{T}	Japan	Gut of abalone	2005	DQ922915
31	*Vibrio coralliilyticus*	LMG 20984^{T}	Indian Ocean	Coral	1999	AJ440005
32	*Vibrio cortegadensis*	LMG 27474^{T}	Spain	Venerupis decussata	2004/2005	HF955040
33	*Vibrio crassostreae*	LGP 7^{T}	France	Atlantic coast	2001	EF094887
34	*Vibrio crosai*	CAIM 1437^{T}	Mexico	Crassostrea gigas (oyster)	2003	JQ434120
35	*Vibrio cyclitrophicus*	P-2P44^{T}	USA	Seabed sediment	-	AM162656
36	*Vibrio diabolicus*	LMG 19805^{T}	East Pacific	Pompeii worm	1999	X99762
37	*Vibrio diazotrophicus*	ATCC 33466^{T}	Canada	Gut of sea urchin	1981	X74701
38	*Vibrio europaeus*	PP-638^{T}	Spain	Shellfish hatchery	2001	AY792622
39	*Vibrio ezurae*	LMG 19970^{T}	Japan	Gut of abalone	1999	AY426980
40	*Vibrio fluvialis*	ATCC 33809^{T}	Bangladesh	Patient	1976	X74703
41	*Vibrio fortis*	CAIM 629^{T}	Ecuador	Vannamei larva	1996	AJ514916
42	*Vibrio fujianensis*	FJ201301^{T}	China	Aquaculture water	2013	KY951464
43	*Vibrio furnissii*	ATCC 35016^{T}	Japan	Human feces	1969	X76336
44	*Vibrio galatheae*	S2757^{T}	Australia	Mussel	2007	FJ457478
45	*Vibrio gallaecicus*	CECT 7244^{T}	Spain	Ruditapes philippinarum	2004/2005	EU541605
46	*Vibrio gallicus*	CIP 107863	France	Abalone	2001	AY257972
47	*Vibrio gazogenes*	ATCC 29988^{T}	USA	Saltwater marsh	1978	X74705
48	*Vibrio gigantis*	LGP 13^{T}	France	Cultured oysters	2005	EF094888
49	*Vibrio halioticoli*	IAM 14596^{T}	Japan	Gut of abalone	1991	AB000390

50	*Vibrio hangzhouensis*	cn83^T	China	Seabed sediment	-	EU082035
51	*Vibrio harveyi*	NCIMB1280^T	USA	Dead amphip	1935	AY750575
52	*Vibrio hemicentroti*	AlyHP32	South Korea	Gut of Hemicentrotus pulcherrimus	2011	JX204734
53	*Vibrio hepatarius*	LMG 20362^T	Ecuador	Litopenaeus vannamei	1996	AJ345063
54	*Vibrio hippocampi*	BFLP-4^T	Spain	Intestinal content of Hippocampus guttulatus	-	FN421434
55	*Vibrio hispanicus*	LMG 13240^T	Spain	Artemia sp.	1991	AY254039
56	*Vibrio hyugaensis*	090810a^T	Japan	Seawater	2010	LC004912
57	*Vibrio ichthyoenteri*	ATCC 700023^T	Japan	Intestine of japanese flounder larvae	-	AJ421445
58	*Vibrio inhibens*	BFLP-10^T	Japan	Hippocampus guttulatus	-	FN687911
59	*Vibrio injenensis*	KCTC 32233^T	South Korea	Human blood	2015	KC634073
60	*Vibrio inusitatus*	RW14^T	USA	Gut of abalone	2005	DQ922920
61	*Vibrio ishigakensis*	JCM 19231^T	Japan	Seawater	-	KP790249
62	*Vibrio jasicida*	CAIM 1864^T	New Zealand	Haemolymph of Rock lobster	1999	AB562589
63	*Vibrio kanaloae*	LMG 20539^T	France	Oyster larva	1998	AM162657
64	*Vibrio lentus*	CECT 5110^T	Spain	Oyster	-	AJ278881
65	*Vibrio litoralis*	DSM 17657^T	South Korea	Coastal seawater	-	DQ097523
66	*Vibrio madracius*	A-354^T	Brazil	Coral	-	KC751062
67	*Vibrio mangrovi*	MSSRF38^T	India	mangrove-associated wild rice	2009	EU144014
68	*Vibrio marisflavi*	CECT 7928^T	China	Seawater	2008	FJ847833
69	*Vibrio maritimus*	R-40493^T	Brazil	Coral	2005	GU929925
70	*Vibrio mediterranei*	50^T	Spain	Seawater	-	X74710
71	*Vibrio metoecus*	OP3H^T	USA	Saline pond	2006	KJ647312
72	*Vibrio metschnikovii*	JCM 21189^T	Asia	Sick poultry	1888	X74711
73	*Vibrio mexicanus*	CAIM 1540^T	Mexico	Oyster	2004	JQ434105
74	*Vibrio mimicus*	ATCC 33653^T	USA	Patient's ear	-	X74713
75	*Vibrio mytili*	CECT 632^T	Spain	Mussel	1985	X99761

76	*Vibrio natriegens*	NBRC 15636^T	USA	Saltwater marsh	-	X74714
77	*Vibrio navarrensis*	CIP 103381^T	Spain	Sewage	1982	X74715
78	*Vibrio neonatus*	HDD3-1^T	Japan	Gut of Japanese abalones	1999	AY426979
79	*Vibrio neptunius*	LMG 20536	Brazil	Scallop hatchery	1998	AJ316171
80	*Vibrio nereis*	ATCC 25917^T	USA	Seawater	-	X74716
81	*Vibrio nigripulchritudo*	ATCC 27043^T	USA	Seawater	-	X74717
82	*Vibrio ordalii*	ATCC 33509^T	USA	Oncorhynchus kisutch	1973	X74718
83	*Vibrio orientalis*	ATCC 33934^T	China	Seawater	-	X74719
84	*Vibrio ostreicida*	PP-203^T	Spain	Ostrea edulis	-	AJ296159
85	*Vibrio owensii*	CAIM 1854^T	Australia	Diseased larvae	2007	GU018180
86	*Vibrio pacinii*	LMG 19999^T	China	Chinese shrimp	1996	AJ316194
87	*Vibrio pacinii*	MSSRF3^T	India	Mangrove-associated wild rice	2006	DQ847123
88	*Vibrio palustris*	EAod9^T	Spain	Arthrocnemum macrostachyum	2014	KU320862
89	*Vibrio panuliri*	LBS2^T	Indian Ocean, Andaman	Panuliruspeniciliatus	2012	KF487035
90	*Vibrio parahaemolyticus*	ATCC 17802^T	Japan	Patient	1950	AF388386
91	*Vibrio pectenicida*	A365^T	France	Pecten maximus	1991	Y13830
92	*Vibrio penaeicida*	DSM 14398^T	Japan	Penaeus japonicus (Kuruma shrimp)	1994	AJ421444
93	*Vibrio ponticus*	CECT 5869^T	Spain	Cultured mussel	1986	AJ630103
94	*Vibrio porteresiae*	DSM 19223^T	India	Porteresia coarctata	2006	EF488079
95	*Vibrio proteolyticus*	ATCC 15338^T	USA	Intestinal content of louse	-	X74723
96	*Vibrio quintilis*	CECT 7734^T	Spain	Coastal seawater	2008	HE613736
97	*Vibrio renipiscarius*	CECT 8603^T	Spain	Sparus aurata	2000	HG931125
98	*Vibrio rotiferianus*	CAIM 577^T	Belgium	Rotifer from water	1999	AJ316187
99	*Vibrio ruber*	VR1^T	China，Taiwan	Seawater	2011	AF462458
100	*Vibrio rumoiensis*	S-1^T	Japan	waste water	1999	AB013297

101	*Vibrio sagamiensis*	NBRC 104589^T	Japan	Seawater	2005	AB428909
102	*Vibrio salilacus*	DSG-S6^T	China	Saltwater lake	2013	KP234045
103	*Vibrio scophthalmi*	LMG 19158^T	Spain	Scophthalmus maximus	1990	HM771340
104	*Vibrio sinaloensis*	CAIM 797^T	Mexico	Spotted rose snapper spleen	2003	DQ451211
105	*Vibrio sonorensis*	CAIM 1076^T	Mexico	Crassostrea gigas	2003	KT732014
106	*Vibrio spartinae*	SMJ21^T	Spain	Heavy metal polluted estuary	2013	KX583607
107	*Vibrio splendidus*	LMG 4042^T	-	Fish	-	AJ515230
108	*Vibrio superstes*	G3-29^T	Australia	Abalone	2000	AY155585
109	*Vibrio tapetis subsp. britannicus*	CETE 8161^T	UK	Hippoglossus hippoglossus	2001	HE795148
110	*Vibrio tapetis subsp. tapetis*	CECT4600^T	France	Venerupis philippinarum	1990	Y08430
111	*Vibrio tasmaniensis*	LMG 20012^T	Australia	Fish	-	AJ316192
112	*Vibrio thalassae*	CECT 8203^T	Mediterranean Sea	Seawater	2008	HF541973
113	*Vibrio toranzoniae*	CECT 7225^T	Spain	Clams of Ruditapes decussatus	2004	HE978310
114	*Vibrio tritonius*	AM2^T	Japan	Gut of marine invertebrate	2008	GU951698
115	*Vibrio tubiashii*	ATCC 19109^T	USA	Clams	1965	X74725
116	*Vibrio variabilis*	R-40492^T	Brazil	Mucus	2005	GU929924
117	*Vibrio vulnificus*	ATCC 27562^T	USA	Human blood	1979	X76333
118	*Vibrio xiamenensis*	G21^T	China	Soil	2007	GQ397859
119	*Vibrio xuii*	DSM 17185^T	China	Shrimp culture water	1995	AJ316181
120	*Aeromonas hydrophila*	ATCC 7966^T	-	-	-	AY264937

Table S2: Genomic overview of *Vibrio* species analyzed in this study

No.	Species	Strain	Genome Size	GC content (%)	Isolation Region	Isolation Year	Accession Number
1	*Vibrio anguillarum*	NCTC 12159^{T}	4.42	44.40	USA	1900/1988	PRJEB6403
2	*Vibrio bivalvicida*	605^{T}	4.92	44.30	Spain	2012	PRJNA297724
3	*Vibrio cholerae*	O1 biovar El Tor str. N16961^{T}	4.03	47.49	Bangladesh	1971	PRJNA36
4	*Vibrio cholerae*	O139 serovar MO45	4.02	47.50	India	1992	PRJNA242443
5	*Vibrio cincinnatiensis*	NCTC 12012^{T}	3.67	43.70	USA	-	PRJEB19569
6	*Vibrio diazotrophicus*	NBRC 103148^{T}	4.72	43.40	Canada	-	PRJDB1326
7	*Vibrio europaeus*	PP-638^{T}	5.47	44.99	Spain	2001	PRJNA312891
8	*Vibrio fujianensis*	FJ201301^{T}	3.62	43.40	China	2013	PRJNA381276
9	*Vibrio hyugaensis*	090810a^{T}	5.61	44.99	Japan	2010	PRJNA429390
10	*Vibrio injenensis*	KCTC 32233^{T}	3.67	44.00	South Korea	2015	PRJDB5318
11	*Vibrio jasicida*	CECT 7692^{T}	5.99	45.10	-	-	PRJNA427410
12	*Vibrio metschnikovii*	JCM 21189^{T}	3.75	44.19	-	1888	PRJNA40493
13	*Vibrio owensii*	CAIM 1854^{T}	6.35	43.90	Australia	2007	PRJNA189697
14	*Vibrio pacinii*	DSM 19139^{T}	4.30	45.20	China	-	PRJNA234841
15	*Vibrio parahaemolyticus*	RIMD 2210633^{T}	5.15	45.40	Japan	1950	PRJNA233509
16	*Vibrio salilacus*	DSG-S6^{T}	3.62	45.10	China	2013	PRJNA390014
17	*Vibrio vulnificus*	ATCC 27562^{T}	5.01	46.71	USA	1979	PRJNA295776

Table S3. Virulence-associated factors profile of *V. fujianensis* and other pathogenic *Vibrio* species.

VF class	Virulence factors	Related genes	A	B	C	D	E	F	G	H	I	J	K	L
Adherence	Accessory colonization factor	*acfA*	-	-	-	-	-	-	-	+	-	-	-	-
		acfB	+	-	-	-	-	-	-	+	+	-	-	+
		acfC	-	-	-	-	-	-	-	+	+	-	-	-
		acfD	-	-	-	-	-	-	-	+	+	-	-	-
	Mannose-sensitive hemagglutinin (MSHA type IV pilus)	*mshA*	+	+	+	-	-	+	-	+	-	-	+	+
		mshB	-	+	+	-	+	+	+	+	+	-	+	+
		mshC	+	+	+	-	-	+	+	+	+	-	+	+
		mshD	+	+	+	-	+	-	+	+	+	-	+	+
		mshE	+	+	+	+	+	+	+	+	+	+	+	+
		mshF	-	+	+	-	-	-	-	+	+	-	+	-
		mshG	+	+	+	+	+	+	+	+	+	+	+	+
		mshH	+	+	+	+	+	+	+	+	+	+	+	+
		mshI	+	+	+	+	-	+	+	+	+	+	+	+
		mshJ	-	+	+	+	+	+	+	+	+	+	+	+
		mshK	-	+	+	-	-	+	+	+	+	-	+	-
		mshL	+	+	+	+	+	+	+	+	+	+	+	+
		mshM	+	+	+	+	+	+	+	+	+	+	+	+
		mshN	-	+	+	+	-	+	+	+	+	-	+	-
	Toxin-coregulated pilus	*tcpA*	-	-	-	-	-	-	-	+	+	-	-	-

(type IVB pilus)	*tcpB*	-	-	-	-	-	-	-	+	+	-	-	-
	tcpC	-	-	-	-	-	-	-	+	+	-	-	-
	tcpD	-	-	-	-	-	-	-	+	+	-	-	-
	tcpE	-	-	-	-	-	-	-	+	+	-	-	-
	tcpF	-	-	-	-	-	-	-	+	+	-	-	-
	tcpH	-	-	-	-	-	-	-	+	+	-	-	-
	tcpI	-	-	-	-	-	-	-	+	+	-	-	-
	tcpJ	-	-	-	-	-	-	-	+	+	-	-	-
	tcpN/toxT	-	-	-	-	-	-	-	+	+	-	-	-
	tcpP	-	-	-	-	-	-	-	+	+	-	-	-
	tcpQ	-	-	-	-	-	-	-	+	+	-	-	-
	tcpR	-	-	-	-	-	-	-	+	+	-	-	-
	tcpS	-	-	-	-	-	-	-	+	+	-	-	-
	tcpT	-	-	-	-	-	-	-	+	+	-	-	-
	pilA	+	-	+	-	+	-	+	+	+	-	+	-
Type IV pilus	*pilB*	+	+	+	+	+	+	+	+	+	+	+	+
	pilC	+	+	+	+	+	+	+	+	+	+	+	+
	pilD	+	+	+	+	+	+	+	+	+	+	+	+
	wbpE	-	-	-	-	-	-	-	-	-	+	-	-
	wbpG	-	-	-	-	-	-	-	-	-	+	-	-
LPS O-antigen	*wbpH*	-	-	-	-	-	-	-	-	-	+	-	-
	wbpI	-	-	-	-	-	-	-	-	-	+	-	-
	Undetermined	-	-	-	-	+	-	-	-	-	-	-	-
	Undetermined	+	-	-	+	-	-	+	-	+	-	-	-
Flp type IV pili	*flpF*	-	-	-	-	-	+	+	-	-	-	-	+
The tad locus	*tadA*	+	-	-	-	-	-	-	-	-	-	-	+

	Lateral flagella	*flgI*	-	-	-	-	-	+	-	-	-	-	-	-
		lfgH	-	-	-	-	-	+	-	-	-	-	-	-
		lfhA	-	-	-	-	-	+	-	-	-	-	-	-
	Polar flagella	*nueB*	-	+	-	-	-	-	-	-	-	-	-	-
	Curli fibers	*csgG*	-	-	-	-	-	+	+	-	-	-	-	-
Antiphagocytosis	Capsular polysaccharide	*cpsA*	-	+	+	+	-	-	-	+	+	+	+	-
		cpsB	-	+	+	+	-	-	-	+	+	+	+	-
		cpsC	-	+	+	-	-	-	-	+	+	-	+	-
		cpsD	-	+	+	+	-	-	-	+	+	+	+	-
		cpsE	-	-	+	-	-	-	-	-	-	-	+	-
		cpsF	-	+	+	+	-	-	-	+	+	+	+	-
		cpsG	-	-	+	-	-	-	-	-	-	-	+	-
		cpsH	-	+	+	-	-	-	-	-	-	-	+	-
		cpsI	-	+	+	-	-	-	-	-	-	-	+	-
		cpsJ	-	+	+	-	-	-	-	-	-	-	+	-
		rmlA	-	-	-	-	+	-	-	-	-	-	-	-
		rmlC	-	+	-	-	-	-	-	-	-	-	-	-
		rmlD	-	+	-	-	-	-	+	-	-	-	-	-
		wbfB	-	-	-	+	-	-	-	-	+	+	-	-
		wbfT	+	-	-	-	-	-	-	-	-	-	-	-
		wbfU	+	-	-	-	+	-	-	+	+	-	-	-
		wbfV/wcvB	+	+	+	+	+	+	+	+	+	-	+	+
		wbfY	+	-	+	-	+	-	-	+	+	-	-	+
		wbjD/wecB	-	-	-	+	-	-	-	+	+	+	-	-
		wecA	-	+	-	-	-	+	+	-	-	-	+	-
		wecC	-	-	-	+	-	-	-	+	+	+	-	-

		wza	-	+	+	+	-	+	+	-	-	-	+	-
		wzb	-	+	-	-	-	+	-	-	-	-	+	-
		wzc	-	+	+	+	-	+	+	-	-	-	+	-
	Capsule	*uge*	-	-	-	-	-	+	+	-	-	-	-	-
		cheA	+	+	+	+	+	+	+	+	+	+	+	+
		cheB	+	+	+	+	+	+	+	+	+	+	+	+
		cheR	+	+	+	+	+	+	+	+	+	+	+	+
		cheV	+	+	+	+	-	+	+	+	+	+	+	+
		cheW	+	+	+	-	+	+	+	+	+	-	+	+
		cheY	+	+	+	-	+	+	+	+	+	+	+	+
		cheZ	+	+	+	+	+	+	+	+	+	+	+	+
		filM	+	+	+	+	+	+	+	+	+	+	+	+
		flaA	+	+	+	+	+	+	+	+	+	+	+	+
		flaB	+	+	+	+	+	+	+	+	+	+	+	+
Chemotaxis and motility	Flagella	*flaC*	-	-	-	+	-	+	+	+	+	+	-	-
		flaD	+	+	+	+	+	+	+	+	+	+	+	+
		flaE	+	+	+	+	+	+	+	+	+	+	+	+
		flaG	+	+	+	-	+	+	+	+	+	-	+	+
		flaI	+	+	+	-	+	+	+	+	+	-	+	+
		flgA	+	+	+	+	+	+	+	+	+	+	+	+
		flgB	+	+	+	-	+	+	+	+	+	-	+	+
		flgC	+	+	+	-	+	+	+	+	+	-	+	+
		flgD	+	+	+	+	+	+	+	+	+	+	+	+
		flgE	+	+	+	+	+	+	+	+	+	+	+	+
		flgF	+	+	+	+	+	+	+	+	+	+	+	+
		flgG	+	+	+	+	+	+	+	+	+	+	+	+

flgH	+	+	+	+	+	+	+	+	+	+	+	+
flgI	+	+	+	+	+	+	+	+	+	+	+	+
flgJ	+	+	+	+	+	+	+	+	+	+	+	+
flgK	+	+	+	+	+	+	+	+	+	+	+	+
flgL	+	+	+	+	+	+	+	+	+	+	+	+
flgM	+	+	+	-	+	-	-	+	+	-	+	-
flgN	+	+	+	-	+	+	+	+	+	-	+	+
flhA	+	+	+	+	+	+	+	+	+	+	+	+
flhB	+	+	+	+	+	+	+	+	+	+	+	+
flhF	+	+	+	+	+	+	+	+	+	+	+	+
flhG	+	+	+	+	+	+	+	+	+	+	+	+
fliA	+	+	+	+	+	+	+	+	+	+	+	+
fliD	+	+	+	+	+	+	+	+	+	+	+	+
fliE	+	+	+	-	+	+	+	+	+	+	+	+
fliF	+	+	+	+	+	+	+	+	+	+	+	+
fliG	-	+	+	+	+	+	+	+	+	+	+	+
fliH	+	+	+	+	+	+	+	+	+	+	+	+
fliI	+	+	+	+	+	+	+	+	+	+	+	+
fliJ	+	+	+	-	+	+	+	+	+	-	+	+
fliK	-	+	+	+	-	-	-	+	+	-	+	-
fliL	+	+	+	-	+	+	+	+	+	-	+	+
fliN	+	+	+	-	+	+	+	+	+	-	+	+
fliO	+	+	+	-	-	+	+	+	+	-	+	-
fliP	+	+	+	+	+	+	+	+	+	+	+	+
fliQ	+	+	+	-	+	+	+	+	+	+	+	+
fliR	+	+	+	+	+	+	+	+	+	+	+	+

		fliS	+	+	+	-	+	+	+	+	+	-	+	+
		flrA	+	+	+	+	+	+	+	+	+	+	+	+
		flrB	+	+	+	+	+	+	+	+	+	+	+	+
		flrC	+	+	+	+	+	+	+	+	+	+	+	+
		motA	+	+	+	+	+	+	+	+	+	+	+	+
		motB	+	+	+	+	+	+	+	+	+	+	+	+
		motX	+	+	+	+	+	+	+	+	+	+	+	+
		motY	+	+	+	+	+	+	+	+	+	+	+	+
Enzyme	Metalloproteases	*hap/vvp*	-	+	-	+	-	+	+	+	+	+	-	-
	Neuraminidase	*nanH*	-	-	-	+	-	-	-	+	-	+	-	-
Iron uptake	Enterobactin receptors	*irgA*	+	-	+	+	-	+	+	+	+	+	+	+
		vctA	+	-	+	+	-	+	+	+	+	-	+	+
	Heme receptors	*hasR*	-	-	-	-	+	-	-	+	-	-	-	-
		hutA	-	+	+	+	+	+	+	+	+	+	+	-
		hutR	-	+	+	-	-	-	-	+	+	-	+	-
	Periplasmic binding protein-dependent ABC transport systems	*vctC*	+	+	+	+	+	+	+	+	+	+	+	+
		vctD	+	+	+	+	+	+	+	+	+	+	+	+
		vctG	+	+	+	+	+	+	+	+	+	+	+	+
		vctP	+	+	+	+	+	+	+	+	+	+	+	+
		viuC	+	-	-	-	+	+	+	+	+	-	+	+
		viuD	+	-	-	-	-	+	+	+	+	-	-	+
		viuG	+	-	-	-	-	+	+	+	+	-	-	+
		viuP	+	-	-	-	-	+	+	+	+	-	-	+
	Vibriobactin biosynthesis	*vibA*	+	+	-	-	+	+	+	+	+	+	+	+
		vibB	+	+	-	-	+	+	+	+	+	+	+	+
		vibC	+	+	-	-	+	+	+	+	+	+	+	+

		vibD	-	+	-	-	-	-	-	+	+	-	-	-
		vibE	+	+	-	-	+	+	+	+	+	+	+	+
		vibF	-	+	-	-	-	-	-	+	+	-	-	-
		vibH	-	+	-	-	-	-	-	+	+	-	-	-
	Vibriobactin utilization	*viuA*	-	+	-	-	-	-	-	+	+	-	-	-
		viuB	-	+	-	-	-	-	-	+	+	-	-	-
		barB	-	-	-	-	-	-	-	-	-	+	+	-
		bauB	-	-	-	-	-	-	-	-	-	+	+	-
	Acinetobactin	*bauC*	-	-	-	-	-	-	-	-	-	+	+	-
		bauD	-	-	-	-	-	-	-	-	-	+	+	-
		basG	+	-	-	-	-	-	-	-	-	-	-	-
		fepB	-	-	-	-	-	-	-	-	-	-	+	-
	Enterobactin transport	*fepD*	-	-	-	-	-	-	-	-	-	-	+	-
		fepG	-	-	-	-	+	-	-	-	-	-	-	-
	ABC transporter	*fagA*	-	-	-	-	-	+	-	-	-	-	-	-
		fagB	-	-	-	-	-	+	-	-	-	-	-	-
Quorum sensing	Autoinducer-2	*luxS*	+	+	+	-	+	+	+	+	+	-	+	+
	Cholerae autoinducer-1	*cqsA*	-	-	+	+	+	+	+	+	+	+	+	+
		epsC	+	+	+	+	+	+	+	+	+	+	+	+
		epsE	+	+	+	+	+	+	+	+	+	+	+	+
		epsF	+	+	+	+	+	+	+	+	+	+	+	+
Secretion system	EPS type II secretion system	*epsG*	+	+	+	-	+	+	+	+	+	-	+	+
		epsH	+	+	+	-	+	+	+	+	+	+	+	+
		epsI	+	+	+	-	+	+	+	+	+	-	+	+
		epsJ	+	+	+	+	+	+	+	+	+	+	+	+
		epsK	+	+	+	+	+	+	+	+	+	+	+	+

	epsL	+	+	+	+	+	+	+	+	+	+	+	+
	epsM	+	+	+	-	-	+	+	+	+	-	+	+
	epsN	+	+	+	+	+	+	+	+	+	-	+	-
	gspD	+	+	+	+	+	+	+	+	+	+	+	+
	Undetermined	-	-	+	-	-	-	-	-	-	-	-	-
T3SS1 secreted effectors	*vopQ*	-	-	+	-	-	-	-	-	-	-	+	-
	vopR	-	-	+	-	-	-	-	-	-	-	+	-
	vopS	-	-	+	-	-	-	-	-	-	-	+	-
	sycN	-	-	+	-	-	-	-	-	-	-	+	-
	tyeA	-	-	+	-	-	-	-	-	-	-	+	-
	vcrD	-	-	+	-	-	-	-	-	-	-	+	-
	vcrG	-	-	+	-	-	-	-	-	-	-	+	-
	vcrH	-	-	+	-	-	-	-	-	-	-	+	-
	vcrR	-	-	+	-	-	-	-	-	-	-	+	-
	vcrV	-	-	+	-	-	-	-	-	-	-	+	-
	virF	-	-	+	-	-	-	-	-	-	-	+	-
T3SS1	*virG*	-	-	+	-	-	-	-	-	-	-	+	-
	vopB	-	-	+	-	-	-	-	-	-	-	+	-
	vopD	-	-	+	-	-	-	-	-	-	-	+	-
	vopN	-	-	+	-	-	-	-	-	-	-	+	-
	vscA	-	-	+	-	-	-	-	-	-	-	+	-
	vscB	-	-	+	-	-	-	-	-	-	-	+	-
	vscC	-	-	+	-	-	-	-	-	-	-	+	-
	vscD	-	-	+	-	-	-	-	-	-	-	+	-
	vscF	-	-	+	-	-	-	-	-	-	-	+	-
	vscG	-	-	+	-	-	-	-	-	-	-	+	-

	vscH	-	-	+	-	-	-	-	-	-	-	+	-
	vscI	-	-	+	-	-	-	-	-	-	-	+	-
	vscJ	-	-	+	-	-	-	-	-	-	-	+	-
	vscK	-	-	+	-	-	-	-	-	-	-	+	-
	vscL	-	-	+	-	-	-	-	-	-	-	+	-
	vscN	-	-	+	-	-	-	-	-	-	-	+	-
	vscO	-	-	+	-	-	-	-	-	-	-	+	-
	vscP	-	-	+	-	-	-	-	-	-	-	-	-
	vscQ	-	-	+	-	-	-	-	-	-	-	+	-
	vscR	-	-	+	-	-	-	-	-	-	-	+	-
	vscS	-	-	+	-	-	-	-	-	-	-	+	-
	vscT	-	-	+	-	-	-	-	-	-	-	+	-
	vscU	-	-	+	-	-	-	-	-	-	-	+	-
	vscX	-	-	+	-	-	-	-	-	-	-	+	-
	vscY	-	-	+	-	-	-	-	-	-	-	+	-
	vxsC	-	-	+	-	-	-	-	-	-	-	+	-
	vopA	-	-	+	-	-	-	-	-	-	-	-	-
T3SS2 secreted effectors	*vopC*	-	-	+	-	-	-	-	-	-	-	-	-
	vopL	-	-	+	-	-	-	-	-	-	-	-	-
	vopT	-	-	+	-	-	-	-	-	-	-	-	-
	Undetermined	-	-	+	-	-	-	-	-	-	-	-	-
	Undetermined	-	-	+	-	-	-	-	-	-	-	-	-
T3SS2	Undetermined	-	-	+	-	-	-	-	-	-	-	-	-
	Undetermined	-	-	+	-	-	-	-	-	-	-	-	-
	Undetermined	-	-	+	-	-	-	-	-	-	-	-	-
	Undetermined	-	-	+	-	-	-	-	-	-	-	-	-

		Undetermined	-	-	+	-	-	-	-	-	-	-	-	-
		Undetermined	-	-	+	-	-	-	-	-	-	-	-	-
		vcrD2	-	-	+	-	-	-	-	-	-	-	-	-
		vscC2	-	-	+	-	-	-	-	-	-	-	-	-
		vscN2	-	-	+	-	-	-	-	-	-	-	-	-
		hcp-2	-	-	-	-	-	+	+	+	+	-	-	-
	VAS effector proteins	*vgrG-1*	-	-	-	-	-	-	-	+	+	+	-	-
		vgrG-2	-	-	-	+	-	+	+	+	+	+	-	-
		vgrG-3	-	-	-	+	-	-	+	+	+	+	-	-
		vasA	-	-	-	+	-	+	+	+	+	+	-	-
		vasB	-	-	-	+	-	+	+	+	+	+	-	-
		vasC	-	-	-	+	-	+	+	+	+	+	-	-
		vasD	-	-	-	-	-	+	+	+	+	-	-	-
		vasE	-	-	-	+	-	+	+	+	+	+	-	-
	VAS type VI secretion system	*vasF*	-	-	-	+	-	+	+	+	+	+	-	-
		vasG	-	-	-	+	-	+	+	+	+	+	-	-
		vasH	-	-	-	+	-	+	+	+	+	+	-	-
		vasI	-	-	-	+	-	+	+	+	+	+	-	-
		vasJ	-	-	-	+	-	+	+	+	+	+	-	-
		vasK	-	-	-	+	-	+	+	+	+	+	-	-
	T4SS effectors	Undetermined	-	-	-	-	-	-	-	-	-	-	+	-
	Accessory cholera enterotoxin	*ace*	-	-	-	-	-	-	-	+	+	-	-	-
	Cholera toxin	*ctxA*	-	-	-	-	-	-	-	+	+	-	-	-
Toxin	Hemolysin/cytolysin	*vvhA*	-	+	-	-	-	-	-	-	-	-	-	-
	RTX toxin	*rtxA*	-	+	-	-	-	-	-	+	+	+	-	-
		rtxB	-	+	-	-	-	-	-	+	+	+	-	-

		rtxC	-	+	-	-	-	-	-	+	+	-	-	-
		rtxD	-	+	-	-	-	-	-	+	+	+	-	-
		rtxE	-	+	-	-	-	-	-	-	-	-	-	-
	Thermolabile hemolysin	*tlh*	-	+	+	+	+	+	+	+	+	+	+	-
	Thermostable direct hemolysin	*tdh*	-	-	+	-	-	-	-	-	-	-	-	-
	V.cholerae cytolysin	*hlyA*	-	-	-	+	+	+	+	+	+	+	-	-
	Zona occludens toxin	*zot*	-	-	-	-	-	-	-	+	+	-	-	-
	Phytotoxin coronatine	Undetermined	-	-	-	-	-	+	+	-	-	-	+	-
Others	O-antigen	Undetermined	+	-	-	-	-	-	-	-	+	-	-	-
		manB	-	-	-	-	-	+	+	-	-	-	-	+
Endotoxin	LOS	*lgtF*	+	-	-	-	+	+	+	-	-	-	-	+
		kdkA	-	-	-	-	+	+	+	-	-	-	-	-
		lpxK	-	-	-	-	+	-	-	-	-	+	-	-
		opsX/rfaC	+	-	-	-	+	-	-	-	-	-	-	-
		rfaF	-	-	-	-	+	-	-	-	-	-	-	-
	LPS	*bplA*	-	-	-	-	-	-	-	-	-	+	-	-
Immune evasion	Capsule	Undetermined	-	+	-	+	+	+	+	-	-	+	+	-
Invasion	Flagella	*cheB2*	+	-	-	-	+	+	-	-	-	+	-	+
Iron acquision	Ent siderophore	Undetermined	-	-	-	-	+	-	-	-	-	+	+	-
		Undetermined	-	-	-	-	+	-	-	-	-	-	-	-
		fepG	-	-	-	-	-	-	-	-	-	-	+	-
Biofilm formation	AdeFGH efflux pump/transport autoinducer	*adeG*	-	-	-	-	-	-	-	-	-	-	+	-
Cell surface components	Trehalose-recycling ABC transporter	*sugC*	-	-	-	-	-	+	-	-	-	-	-	+

Regulation	Two-component system	*bvgA*	-	-	-	-	-	-	-	-	-	-	-	+
		bfmR	+	-	-	-	+	-	-	-	-	-	-	-
Anaerobic respiration	Nitrate reductase	*narH*	-	-	-	-	-	-	+	-	-	-	-	-
Efflux pump	AcrAB	Undetermined	-	-	-	-	-	-	+	-	-	-	-	-
		acrB	-	-	-	-	-	+	-	-	-	-	-	-

Note: A. *Vibrio fujianensis* FJ201301^{T}; B. *Vibrio vulnificus* ATCC 27562T ; C. *Vibrio parahaemolyticus* RIMD 2210633^{T}; D. *Vibrio mimicus* NCTC 11435^{T}; E. *Vibrio metschnikovii* JCM 21189^{T}; F. *Vibrio furnissii* NCTC 13120^{T}; G. *Vibrio fluvialis* ATCC 33809^{T}; H. *Vibrio cholerae* O1 biovar El Tor N16961^{T}; I. *Vibrio cholerae* O139 serovar MO45; J. *Vibrio anguillarum* NCTC 12159^{T}; K. *Vibrio alginolyticus* ATCC 17749^{T}; L. *Vibrio cincinnatiensis* NCTC 12012^{T}
